# Risk Factors and Preventive Strategies for Perioperative Distal Femoral Fracture in Patients Undergoing Total Knee Arthroplasty

**DOI:** 10.3390/medicina59020369

**Published:** 2023-02-14

**Authors:** Ki Ho Kang, Man Soo Kim, Jae Jung Kim, Yong In

**Affiliations:** Department of Orthopaedic Surgery, Seoul St. Mary’s Hospital, College of Medicine, The Catholic University of Korea, 222, Banpo-daero, Seocho-gu, Seoul 06591, Republic of Korea

**Keywords:** total knee arthroplasty, femur fracture, polyethylene insert, osteoporosis, multivariate logistic analysis

## Abstract

*Background and Objectives* Perioperative distal femoral fracture is rare in patients undergoing total knee arthroplasty (TKA). In such rare cases, additional fixation might be required, and recovery can be delayed. Several studies have focused on perioperative distal femoral fractures in TKA, but there remains a lack of information on risk factors. The purpose of this study was to investigate risk factors for perioperative distal femoral fractures in patients undergoing TKA and suggest preventive strategies. *Materials and Methods*: This retrospective study included a total of 5364 TKA cases in a single institution from 2011 to 2022. Twenty-four distal femoral fractures occurred during TKA or within one month postoperatively (0.45%). Patient demographics, intraoperative findings, and postoperative progress were obtained from patient medical records and radiographs. Risk factors for fractures were analyzed using multivariate Firth logistic regression analysis. *Results*: Although all 24 distal femoral fractures occurred in female patients (24 of 4819 patients, 0.50%), the incidence rate of fracture between male and female patients was not significantly different (*p* = 0.165). The presence of osteoporosis and insertion of a polyethylene (PE) insert with knee dislocation were statistically significant risk factors (*p* = 0.009 and *p* = 0.046, respectively). However, multivariate logistic regression analysis showed that only osteoporosis with bone mineral density (BMD) < −2.8 (odds ratio (2.30), 95% CI (1.03–5.54), *p* = 0.043) was an independent risk factor for perioperative distal femoral fracture in TKA patients. *Conclusions*: Our results suggest that osteoporosis with BMD < −2.8 is a risk factor for distal femoral fractures in patients undergoing TKA. In these patients, careful bone cutting, adequate gap balancing, and especially the use of the sliding method for insertion of a PE insert are recommended as preventive strategies.

## 1. Introduction

Fracture of the distal femur during primary total knee arthroplasty (TKA) is a rare complication, with two previous studies reporting an overall incidence ranging from 0.39% to 2.2% [1,2]. These fractures can happen intraoperatively or be found during the postoperative rehabilitation period without definite fracture history [3]. Although the majority of studies report an incidence below 1%, if a distal femoral fracture does occur, it can affect postoperative recovery and outcomes [4]. In fractures with minimal displacement, conservative treatment without additional fixation can lead to good outcomes [2]. However, most fractures require additional fixation [5]. Some severe cases require revision TKA [1]. Clinical outcomes are generally good with proper management; however, non-weight bearing or partial weight bearing for a period of 6–8 weeks as well as limitation of range of motion (ROM) after TKA are common, even in minimally displaced cases [4,6].

There are several known risk factors in distal femoral fracture during TKA, including osteoporosis, rheumatoid arthritis (RA), advanced age, female gender, and posterior-stabilized (PS) implant design [1,5,7,8,9,10,11]. Studies have shown that the wide box cut is a risk factor for femoral fracture in PS TKA [8,9]. Recent PS TKA systems apply minimal box-cutting designs, and most PS surgeons would not change their main implant to a cruciate-retaining type in their practice. In other studies reporting on female sex, osteoporosis, and RA as risk factors for femoral fractures during TKA, fewer than 10 cases of femoral fracture were included in each study [5,10,11]. Recently, one study presented risk factors in the Asian population [6], including female sex, osteoporosis, and small femur size; however, objective evaluation of osteoporosis was inadequate because bone mineral density (BMD) measurement was not routinely conducted. There is a lack of research on risk factors for femoral fracture during TKA [1,7].

The purpose of this study was to investigate risk factors of perioperative distal femoral fracture in a TKA series using PS implants at a single institution. We hypothesized that certain patient demographics or surgical techniques might be associated with perioperative distal femoral fracture. We would like to suggest optimal surgical strategies to prevent distal femoral fracture based on risk factors during TKA.

## 2. Materials and Methods

### 2.1. Patient Selection

**Figure 1:**
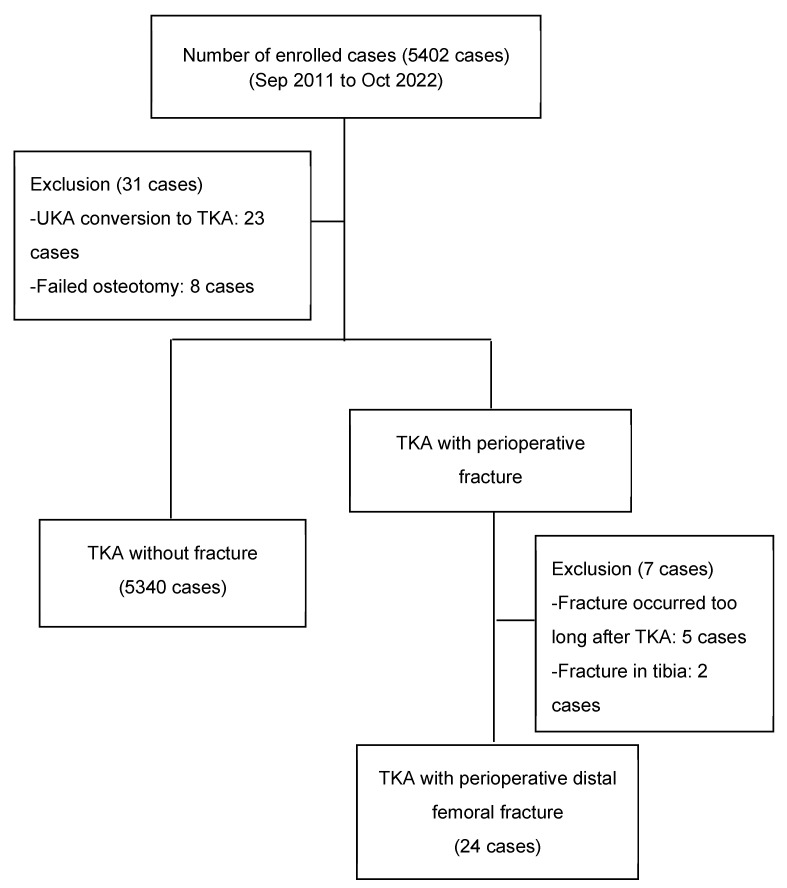
Participant flow diagram.

A total of 5402 primary TKAs was performed on 3922 patients at our institution between September 2011 and October 2022 by two surgeons. All patients were of Asian ethnicity. Patients who previously underwent unicompartmental knee arthroplasty (UKA) and osteotomy around the knee were excluded. A total of 31 fractures occurred during the TKA procedure or within one month after TKA. A patient was excluded if the fracture occurred anywhere other than the distal femur. Cases that had occurred more than one month prior or in which the fracture was caused by definite trauma were also excluded. The remaining 5340 primary TKAs and 24 distal femoral fracture cases were enrolled in this study (Figure 1). This study was approved by the institutional review board of our institution {KC22RASI0865, approved on 25 November 2022}.

### 2.2. Group Assessment

Patients were divided into two groups with or without perioperative distal femoral fracture. The following demographic and surgical factors were compared between the two groups: age, sex, side, body mass index (BMI), preoperative hip-knee-ankle angle (HKAA), preoperative femorotibial angle (FTA), bone mineral density (BMD), surgical interval, type of implant, and mode of polyethylene (PE) insert insertion. All patients underwent BMD evaluation using dual-energy X-ray absorptiometry (DXA, Hologic Inc., Marlborough, MA, USA) prior to surgery. The BMD value was determined as the lowest T-score value among the femur head, total femur, and average lumbar spine, excluding outliers. The surgical interval of TKA was divided into three categories: unilateral TKA, same-day bilateral TKA, and staggered bilateral TKA with a one-week interval. PS-type implants of various knee systems were used, including 2244 TKAs using Lospa (Corentec, Seoul, Republic of Korea), 964 TKAs using Vanguard (Zimmer Biomet, Warsaw, IN, USA), 664 TKAs using advanced coated system (ACS) (Implantcast GmbH, Buxtehude, Germany), 351 TKAs using Exult (Corentec, Seoul, Republic of Korea), 261 TKAs using PFC (DePuy Synthes, Warsaw, IN, USA), 150 TKAs using Persona (Zimmer Biomet, Warsaw, IN, USA), 116 TKAs using Journey II (Smith & Nephew, Memphis, TN, USA), 56 TKAs using Legion (Smith & Nephew, Memphis, TN, USA), and 34 TKAs using Attune (DePuy Synthes, Warsaw, IN, USA). A total of 14 TKAs with fewer than 10 of a particular knee system were not described separately.

### 2.3. Surgical Procedure

**Figure 2:**
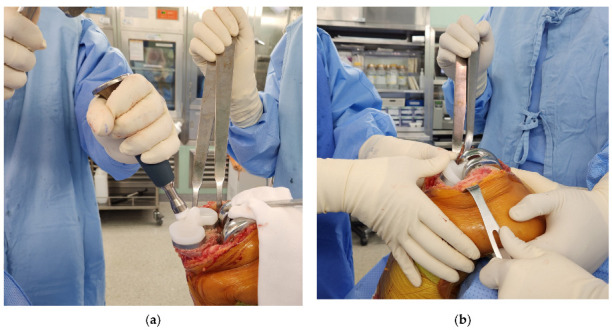
Modes of polyethylene (PE) inert insertion. (**a**) Insertion of the PE insert with knee dislocation. (**b**) Insertion of the PE insert with the sliding method.

All operations were performed by two surgeons in a standard fashion under general anesthesia with tourniquet inflation to 300 mmHg. A PS knee system with the subvastus approach was used in all cases. A measured resection technique was used for bone cutting. Proper gap balancing was applied after bone cutting. Extension and 90° flexion gaps were measured using a tensor device (B.Braun-Aesculap, Tuttlingen, Germany) and scaled forceps (B.Braun-Aesculap) with the application of a 200-N distraction force. In the case of a tight medial gap, multiple needling puncturing was performed with a standard 18-gauge needle based on digital palpation of taut medial collateral ligament (MCL) fibers [12]. All components were fixed using bone cement. Two PE insert insertion methods were used; the knee dislocation method and the sliding method (Figure 2). The PE insert insertion method was determined according to the design of the locking mechanism of the knee system or the status of soft tissue balance. Some knee systems required knee dislocation for secure PE insert insertion, and some allowed sliding insertion of PE insert without knee dislocation.

### 2.4. Statistical Methods

Descriptive analyses were based on means and standard deviations for continuous variables and frequencies and percentages for categorical variables. The chi-square or Fisher exact test for categorical variables was used to compare two groups according to demographic characteristics. In this study, Firth logistic regression analysis was used to estimate the odds ratio (OR) with a 95% confidence interval (CI) due to the small number of fracture cases (24 cases) [13]. Multivariate logistic regression analysis was carried out to identify risk factors significantly related to perioperative distal femoral fracture (*p* < 0.1). All statistical analyses were performed using the SPSS ver. 29.0 program (SPSS Inc., Chicago, IL, USA). A *p*-value < 0.05 was considered statistically significant. Data are expressed as mean values with standard error of the mean (SEM) unless otherwise stated.

## 3. Results

Perioperative distal femoral fractures occurred with an incidence of 0.45% (24 cases) during TKA. Table 1 shows the demographics between patients that underwent TKA without fracture and those with perioperative distal femoral fracture. A total of 17 fractures occurred in 2701 Lospa (Corentec, Seoul, Republic of Korea) TKAs (0.63%) and 7 fractures in 717 ACS (Implantcast GmbH, Buxtehude, Germany) TKAs (0.98%). There were no significant differences between the without fracture group and with fracture group except for BMD value (*p* = 0.009), and mode of PE insert insertion (*p* = 0.046). Although all patients with fractures were female (0.50%), female sex was not a statistically significant factor (*p* = 0.165).

Among the 24 fractures, 22 (91.7%) had a varus knee deformity with an average HKAA of varus of 9.7°. Seventeen fractures (70.8%) happened on the medial femoral condyle of varus deformed knee with an average HKAA of 9.4° (Table 2). The 21 cases (87.5%) that occurred intraoperatively were performed with screw fixation alone. All intraoperative fracture cases but one case achieved bony union, which required revision TKA because subsidence had occurred one month after fixation (Patient B; Table 2). All three postoperative fracture cases occurred 10 to 14 days after the operation without definite trauma history and had a fracture in the lateral femoral condyle that required revision TKA. Among the total 24 fracture patients, 19 (79.2%) were diagnosed with osteoporosis with a BMD value of −2.5 or less, of which 16 cases (66.7%) were identified as osteoporosis with a BMD value less than −2.8.

Based on univariate factor analysis, variables that were statistically different and may affect fracture as potential predictors were used for additional univariate logistic regression analysis. Among them, BMD less than −2.8 (odds ratio [2.30], 95% CI [1.03–5.54], *p* = 0.042) was the only factor that correlated with a risk of perioperative distal femoral fracture in TKA. The BMD value of −2.8 was arbitrarily set as the cut-off value because it was the most statistically significant BMD value among values less than −2.5. BMD value of −2.5 corresponding to osteoporosis was not statistically significant. The parameters included in the multivariate logistic regression model were variables with a *p*-value less than 0.1 and were the mode of PE insert insertion method and BMD less than −2.8. Only one risk factor remained statistically significant. Patients with a BMD value less than −2.8 increased the risk for distal femoral fracture (odds ratio [2.30], 95% CI [1.03–5.54], *p* = 0.043) (Table 3).

## 4. Discussion

This study evaluated the risk factors associated with perioperative distal femoral fractures in primary TKA. Among 5364 TKAs, 24 fractures occurred. The BMD value and mode of PE insert insertion (dislocation method) were the significant risk factors. In multivariate logistic regression analysis of risk factors, only BMD less than −2.8 showed a 2.30-fold increase in fracture risk.

Perioperative distal femoral fracture in TKA is uncommon, and there is a paucity of studies on this topic. Because some fractures that are not clinically important may be missed, the reported incidence might be underestimated [8]. In our study, there was a 0.45% incidence of perioperative femoral fracture with 5364 TKAs, similar to the incidence observed in previous studies (0.39% to 2.2%) [1,2]. According to a recently published systematic review, the overall incidence was from 0.2% to 4.4% [4]. Thus far, prospective studies have not been possible due to the small sample size and lack of predictability of fractures.

The risk factors for distal femoral fracture have been addressed in several previous studies. Osteoporosis is a well-known risk factor, but there is a lack of studies suggesting the cut-off value of osteoporosis. In our institution, DXA was included as a part of the routine preoperative workup for TKA candidates. Most of the patients with fractures were shown to have a low BMD value. In our study, BMD less than −2.8 was the only significant risk factor in multivariate logistic regression analysis. These patients should be informed about their low bone quality and associated risks preoperatively [14]. Then, the surgery should be performed with consent. McClung et al. reported that BMD value is important but emphasized that it was not the only determinant for fracture risk and assessment with individual patient risk [15]. However, according to our study, special attention should be given to patients with a BMD value less than −2.8 during surgery based on the 2.30-fold increase in fracture risk. In our practice, even in patients with osteoporosis, the same rehabilitation program was applied if there was no intraoperative fracture. However, medical treatment for osteoporosis was started or continued depending on the patient’s situation [16].

Alden et al. reported that fracture occurs more commonly in female patients [1]. In our results, the fracture group was all female, which is consistent with previous studies. However, being female was not a significant risk factor in our analysis. According to large population-based cohort studies, an increased risk of symptomatic knee OA was associated with being female, particularly in the Asian population [17]. Although no fracture cases occurred in male patients, since there were more female patients undergoing TKA, the risk of fracture in females was not significantly different in our study.

PS design of TKA has been associated with fracture. Lombardi et al. reported 40 distal femoral intercondylar fractures in 898 PS TKAs (4.4%) [9]. Alden et al. also reported a relative risk of 4.74 for femoral fracture with PS femoral components. In our study, all patients underwent the PS type TKA. Therefore, it was not possible to compare the sample with other types. The wider box cut in the PS type and inappropriate medial or lateral placement to a thin column of bone might result in a high incidence of fracture, which can be reduced by box design improvement and careful resection technique [1,9]. It was expected that advanced age would be a risk factor for fracture, but our results showed no relationship with age. A previous study showed that same-day bilateral TKA was a risk factor, but there was no such correlation in our study based on univariate logistic regression analysis [6]. Other known risk factors, such as chronic use of corticosteroids, RA, or neurological abnormalities, were not included in this analysis because there were few or no corresponding patients [18].

**Figure 3:**
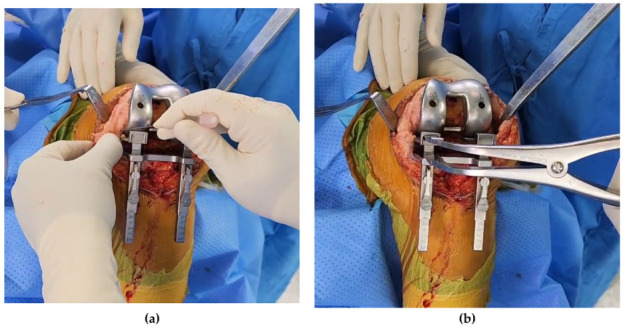
Ease of tension on the medial collateral ligament. (**a**) Pie-crusting with multiple needle puncturing. (**b**) Spreading medial flexion gap using a tensor device.

Perioperative distal femoral fractures can occur at various stages of the TKA procedure. It was reported that most fractures occurred during exposure and bone preparation of the femur and impaction of the femoral implants [1,2]. During the removal of a large osteophyte, inadequate box cut, thin column of the medial and lateral condyle, wrong hammering direction, and/or excessive force could cause fracture at this stage [6,10]. In our study, we found that distal femoral fracture, especially medial femoral condylar fracture, was more likely to occur at a certain stage of TKA. In patients with osteoporosis, we technically focused on soft tissue balancing, especially medial gap, and the PE insert insertion method. A tight medial gap in varus-deformed osteoporotic patients was the most common scenario for distal femoral fracture during TKA at the reduction stage of the knee joint after inserting PE insert with the knee dislocation. Depending on the knee systems, due to the design of the dovetail mechanism, knee dislocation is required to secure the insertion of the PE insert. Greater tension is applied to the MCL with the varus-deformed knee. If the medial gap balancing in the flexion position is not appropriate in these patients, the medial femoral condylar fracture risk is elevated during the knee reduction stage. There were only three cases (12.5%) of fractures among the implants using the sliding method in our study. Although this factor was not significant in our study, it reduced the risk of fracture by 2.73-fold in univariate logistic regression analysis. Alden et al. reported that performing a proper medial release prior to flexing the knee decreased tension on the MCL in the varus knee [1]. Extra attention should be paid to achieving the adequate medial gap, especially when inserting PE insert insertion using the knee dislocation method. After experiencing the fractures, the pie-crusting technique using multiple needle puncturing was applied to obtain the appropriate medial gap for the varus knee in our practice (Figure 3). The pie-crusting technique was reported to be effective and safe for both valgus and varus deformities during TKA [19,20]. In addition, multiple needle puncturing of the MCL is a more effective and safer technique relative to the blade knife technique [12,21].

Management options for perioperative distal femoral fracture are based on stable fixation and on surgeon preference. Huang et al. reported that screw fixation is more appropriate than plate fixation because the reduction of fracture fragments is easy, the plate could have affected the femoral component position, and the cement provides an additional stable force for the fracture fragment [11]. In our study, all fractures found intraoperatively were fixed with a screw alone. Among these cases, only one case of subsidence occurred after one month, and revision TKA was performed in that case. In the three cases of postoperative fractures, revision TKA was also required. These three cases occurred without any definite trauma and were different from previously reported periarticular fractures. The previously published periarticular fracture occurred on average two to four years after TKA with low-energy trauma [22]. It is thought that the perioperative fracture occurred during surgery, but that could not be verified. Shahi et al. reported 15 cases similar to our postoperative fracture cases in fracture patterns, and all of them underwent revision TKA [23]. With the recent development of three-dimensional technology, a clinical workflow for personalized surgical treatment can be developed in postoperative fracture cases [24]. After screw fixation, non-weight bearing or partial weight bearing was recommended to patients for 4–6 weeks, depending on the status of stability. Full weight bearing and full ROM exercise immediately after surgery were allowed in cases of revision TKA.

There exist limitations in our study. First, this study is a retrospective analysis. As mentioned earlier, the low incidence of perioperative fractures with TKA precludes a prospective study. Second, this study was carried out in a single hospital. If the study had included patients from several hospitals, the results would be more relevant, at least at the national level. Third, other known risk factors for distal femoral fracture, such as chronic use of corticosteroids, RA, and neurological abnormalities, could not be included in this analysis because there were few or no corresponding patients. In addition, clinical results confirmed by imaging studies were good in patients with appropriate treatment after a fracture. However, the clinical outcomes were not compared using patient-reported outcome measures (PROMs).

## 5. Conclusions

Perioperative distal femoral fractures during primary TKA are rare, but patients with osteoporotic bone and BMD values less than −2.8 need extra care during the TKA procedure. When inserting the PE insert, the sliding method can reduce the risk of distal femoral fracture. When using the knee dislocation method for insertion of PE insert, the medial and lateral gaps should be sufficiently balanced.

## Figures and Tables

**Table 1:** Demographics of patients with a perioperative distal femoral fracture with TKA.

	TKA without Fracture (*n* = 5340)	TKA with Perioperative Distal Femoral Fracture (*n* = 24)	*p*-Value
Age	70.3 ± 6.6	72.4 ± 7.3	0.125
Sex			0.165
Female	4795 (89.8%)	24 (100%)	
Male	545 (10.2%)	0 (0%)	
Side			0.981
Right	2683 (50.2%)	12 (50%)	
Left	2657 (49.8%)	12 (50%)	
BMI	26.2 ± 6.8	26.3 ± 4.1	0.930
Preoperative HKAA (°)	8.1 ± 5.4 varus	8.4 ± 5.8 varus	0.788
Preoperative FTA (°)	2.9 ± 5.6 varus	2.6 ± 5.1 varus	0.799
BMD	−2.7 ± 1.0	−3.2 ± 0.9	0.009
Type of TKA			0.663
Unilateral TKA	2395 (44.9%)	9 (37.5%)	
Same-Day Bilateral TKA	1810 (33.9%)	10 (41.7%)	
Staggered Bilateral TKA	1135 (21.3%)	5 (20.8%)	
Implant			
Lospa	2684 (50.3%)	17 (70.8%)	
Vanguard	964 (18.1%)	0 (0%)	
ACS	710 (13.3%)	7 (29.2%)	
Exult	351 (6.6%)	0 (0%)	
PFC	261 (4.9%)	0 (0%)	
Persona	150 (2.8%)	0 (0%)	
Journey II	116 (2.2%)	0 (0%)	
Legion	56 (1.0%)	0 (0%)	
Attune	34 (0.6%)	0 (0%)	
Other	14 (0.3%)	0 (0%)	
Mode of PE insert insertion			0.046
Dislocation method	3662 (68.6%)	21 (87.5%)	
Sliding method	1678 (31.4%)	3 (12.5%)	

HKAA; hip-knee-ankle angle; FTA; femorotibial angle.

**Table 2:** Summary of perioperative distal femoral fracture with TKA.

Patient	Sex	Age	Side	BMI	ASA	BMD	Pre FTA (+: Varus, −: Valgus)	Pre HKAA (+: Varus, −: Valgus)	Surgeon	Implant	Mode of PE Insert Insertion	Site of Fracture	Time Discovered	Management
A	F	53	Lt.	25.8	1	−2.7	0.7	7.8	Senior	Lospa	Dislocation	lateral condyle	Intraoperative	Screw fixation
B	F	55	Lt. (Staggered)	22.6	1	−3.6	−10.2	−3.9	Senior	Lospa	Dislocation	medial condyle	Intraoperative	Screw fixation Revision TKA
C	F	69	Rt. (Same-day)	24.2	2	−3.6	5.9	8.2	Senior	Lospa	Dislocation	medial condyle	Intraoperative	Screw fixation
D	F	75	Rt. (Staggered)	24.8	2	−2.9	3.9	9.9	Senior	Lospa	Dislocation	medial condyle	Intraoperative	Screw fixation
E	F	80	Rt.	19.7	2	−3.4	−10.8	−7.5	Senior	Lospa	Dislocation	medial condyle	Intraoperative	Screw fixation
F	F	79	Lt. (Staggered)	30.0	2	−4.4	4.7	12.9	Senior	ACS	Dislocation	medial condyle	Intraoperative	Screw fixation
G	F	74	Lt. (Same-day)	18.7	1	−4.6	9.3	15.5	Senior	Lospa	Dislocation	medial condyle	Intraoperative	Screw fixation
H	F	72	Rt. (Same-day)	25.6	2	−1.9	0.4	2.8	Senior	Lospa	Dislocation	medial condyle	Intraoperative	Screw fixation
I	F	73	Rt.	27.7	2	−3.8	3.1	7.4	Senior	ACS	Dislocation	medial condyle	Intraoperative	Screw fixation
J	F	76	Rt.	23.7	3	−4.1	2.2	10.5	Senior	ACS	Dislocation	medial condyle	Intraoperative	Screw fixation
K	F	73	Rt.	26.7	2	−3.7	0.3	6.1	Senior	ACS	Dislocation	medial condyle	Intraoperative	Screw fixation
L	F	69	Lt. (Same-day)	29.3	2	−2.8	−1.9	5.4	Senior	Lospa	Dislocation	lateral condyle	Postoperative 10 days	Revision TKA
M	F	71	Rt. (Staggered)	35.3	2	−2.2	8.9	16.8	Junior	Lospa	Dislocation	medial condyle	Intraoperative	Screw fixation
N	F	81	Lt.	23.2	3	−3.6	5.1	8.7	Junior	Lospa	Dislocation	medial condyle	Intraoperative	Screw fixation
O	F	65	Lt. (Same-day)	25.2	2	−1.6	8.9	15.1	Senior	ACS	Dislocation	lateral condyle	Intraoperative	Screw fixation
P	F	70	Lt. (Same-day)	33.3	2	−3.7	3.8	12.7	Senior	Lospa	Dislocation	lateral condyle	Postoperative 14 days	Revision TKA
Q	F	66	Lt. (Same-day)	34.6	2	−2.6	8.7	14.1	Senior	Lospa	Dislocation	medial condyle	Intraoperative	Screw fixation
R	F	78	Rt.	26.7	2	−3.1	5.0	6.9	Junior	Lospa	Sliding	medial condyle	Intraoperative	Screw fixation
S	F	78	Rt. (Staggered)	25.5	2	−5.0	2.8	8.2	Senior	ACS	Dislocation	medial condyle	Intraoperative	Screw fixation
T	F	75	Lt. (Same-day)	27.7	2	−2.9	1.1	6.4	Junior	ACS	Dislocation	medial condyle	Intraoperative	Screw fixation
U	F	83	Rt.	25.9	3	−2.1	3.7	14.3	Junior	Lospa	Sliding	medial condyle	Intraoperative	Screw fixation
V	F	77	Lt.	22.6	2	−4.4	5.9	11.5	Senior	Lospa	Dislocation	lateral condyle	Postoperative 14 days	Revision TKA
W	F	74	Rt. (Same-day)	28.8	2	−1.3	0.2	5.2	Junior	Lospa	Sliding	medial condyle	Intraoperative	Screw fixation
X	F	71	Lt. (Same-day)	24.4	2	−2.9	1.1	6.0	Senior	Lospa	Dislocation	medial condyle	Intraoperative	Screw fixation

FASA; American Society of Anesthesiologists, FTA; femorotibial angle, HKAA; hip-knee-ankle angle, F; female, Lt.; left, Rt.; right.

**Table 3:** Multivariate logistic regression analysis of perioperative distal femoral fracture risk factors with TKA.

	Univariate			Multivariate		
	Odds Ratio	95% CI	*p*-Value	Adjusted Odds Ratio	95% CI	*p*-Value
Age	1.05	0.99–1.12	0.127			
Sex (Female)	5.57	0.78–707.69	0.104			
Side (Rt.)	0.99	0.45–2.19	0.981			
Type of TKA						
Unilateral TKA	reference					
Same-Day Bilateral TKA	1.59	0.65–3.96	0.302			
Staggered Bilateral TKA	1.17	0.38–3.27	0.775			
Mode of PE insert insertion (Knee Dislocation)	2.73	0.99–10.25	0.052	1.87	0.68–7.04	0.244
BMD ≤ −2.5 (Osteoporosis)	1.99	0.82–5.71	0.132			
BMD < −2.8	2.30	1.03–5.54	0.042	2.30	1.03–5.54	0.043
Preoperative HKAA	1.01	0.94–1.09	0.800			

HKAA; hip-knee-ankle angle.

## Data Availability

The data published in this research are available on request from the first author (K.H.K).
